# Clozapine but not lithium reverses aberrant tyrosine uptake in patients with bipolar disorder

**DOI:** 10.1007/s00213-023-06397-5

**Published:** 2023-06-15

**Authors:** R Tabrisi, MD Harun-Rashid, J Montero, N Venizelos, M Msghina

**Affiliations:** 1 Department of Plastic Surgery, Faculty of Medicine and Health, Örebro University, Örebro, Sweden; 2 School of Health Sciences, Faculty of Medicine and Health, Örebro University, Örebro, Sweden; 3 School of Medical Sciences, Faculty of Medicine and Health, Örebro University, Örebro, Sweden; 4 Department of Psychiatry, School of Medical Sciences, Faculty of Medicine and Health, Örebro University, Örebro, Sweden; 5 Department of Clinical Neuroscience, Karolinska Institutet, Stockholm, Sweden

**Keywords:** Bipolar disorder, Fibroblasts, Pathophysiology, Clozapine, Lithium, Tyrosine, Dopamine, Noradrenaline

## Abstract

**Rationale:**

Availability of the dopamine and noradrenaline precursor tyrosine is critical for normal functioning, and deficit in tyrosine transport across cell membrane and the blood-brain barrier has been reported in bipolar disorder and schizophrenia. Clozapine and lithium are two psychoactive agents used to treat psychosis, mood disorders and suicidal behavior, but their mechanism of action remains largely unknown.

**Objective:**

To characterize immediate and delayed differences in tyrosine uptake between healthy controls (HC) and bipolar patients (BP) and see if these differences could be normalized by either clozapine, lithium or both. A second objective was to see if clozapine and lithium have additive, antagonistic or synergistic effects in this.

**Method:**

Fibroblasts from five HC and five BP were incubated for 5 min or 6 h with clozapine, lithium, or combination of both. Radioactive labelled tyrosine was used to quantify tyrosine membrane transport.

**Results:**

There was significantly reduced tyrosine uptake at baseline in BP compared to HC, a deficit that grew with increasing incubation time. Clozapine selectively increased tyrosine uptake in BP and abolished the deficit seen under baseline conditions, while lithium had no such effect. Combination treatment with clozapine and lithium was less effective than when clozapine was used alone.

**Conclusions:**

There was significant deficit in tyrosine transport in BP compared to HC that was reversed by clozapine but not lithium. Clozapine was more effective when used alone than when added together with lithium. Potential clinical implications of this will be discussed.

**Supplementary Information:**

The online version contains supplementary material available at 10.1007/s00213-023-06397-5.

## Introduction

Bipolar disorder is a chronic disease characterized by swings of mood with periods of mania and depression, and is known to substantially increase risk for suicide, especially during periods of depression (Miller and Black 2020). Guidelines generally recommend multimodal treatment consisting, among other things, of pharmacological, psychological, and psychosocial interventions, with lithium as the mainstay of pharmacological treatment both during the acute and relapse prevention phases (Baldessarini et al. 2019). Clozapine, which is a first-line treatment option in patients with treatment-resistant psychosis (Okhuijsen-Pfeifer et al. 2018), has also been shown to have significant mood stabilizing properties (Kapczinski et al. 2021). More interestingly, clozapine and lithium are known to reduce suicidal and aggressive behavior and are first-line treatment options for patients with high risk for suicide (Fornaro et al. 2020; Li et al. 2015), making them unique in this among psychoactive drugs (Desai Boström et al. 2023). During recent years, combination treatment with clozapine and lithium is being used increasingly in managing patients with severe self-harm and chronic suicidal behavior (Delgado et al. 2020; Fornaro et al. 2020; Frogley et al. 2012; Kelly et al. 2006; Li et al. 2015), providing further rationale for studying them together. It has been speculated that clozapine and lithium may have a direct effect on suicidal behavior *per se* by reducing suicide-related endophenotypes such as impulsive and aggressive behavior, and not just by treating the underlying affective or psychotic disorder (Kovacsics et al. 2009). However, their cellular mechanism of action remains largely unknown (Foster 2013), and nor is it known whether they have additive or synergistic effects in this.

Dopamine and noradrenaline are two important neurotransmitters with widespread cognitive, affective, and motivation-related functions and are implicated in the pathogenesis and treatment of numerous psychiatric disorders including schizophrenia, depression and bipolar disorder (Arnsten 2007). Both dopamine and noradrenaline have tyrosine as precursor in their rate-limiting step of synthesis (Daubner et al. 2011). In many but not all experimental paradigms, dopamine and noradrenaline synthesis has been shown to increase under behaviorally challenging conditions (Jenson et al. 2015; Kvetnansky et al. 2009; Lehnert and Wurtman 1993), and extracellular tyrosine has been found to enhance dopamine synthesis in neurons actively firing action potential and releasing transmitter substance (Fernstrom and Fernstrom 2007; Lehnert and Wurtman 1993). Under some experimental conditions, acute tyrosine depletion has been found to reduce dopamine synthesis and impair cognitive function (Leyton et al. 2004; Leyton et al. 2000; McTavish et al. 1999; Nagano-Saito et al. 2012; Nagano-Saito et al. 2008). On the other hand, tyrosine supplementation has been shown to enhance cognitive control under stressful and demanding conditions (Jongkees et al. 2015). Studies in individuals at high risk for developing psychosis have also revealed important roles for dopamine synthesis with high-risk individuals who went on to develop psychosis showing significantly higher dopamine synthesis capacity compared to those high-risk individuals who did not develop psychosis (Howes et al. 2011). Tyrosine availability may thus potentially impact cognitive functions and psychiatric disorders related to or modified by catecholaminergic transmission.

Patient-derived skin fibroblasts are increasingly being used as in vitro models for studying physiological and pathophysiological aspects of psychiatric disorders (Kálmán et al. 2016), including schizophrenia (Batalla et al. 2015; Flyckt et al. 2001), depression (Garbett et al. 2015), and bipolar disorder (Persson et al. 2009). A previous study from our group investigating membrane transport of monoamine transmitter precursors in fibroblasts showed that maximal transport velocity (*Vmax*) for tyrosine uptake was significantly lower in patients with bipolar disorder compared to healthy controls (Persson et al. 2009). Studies have also shown that the L-type amino acid transporter 1 (LAT1) is the main transporter of tyrosine across cell membranes in both neural tissue and fibroblasts, and previous studies from our group have shown that 90% of tyrosine transport in human fibroblasts occurs through LAT 1 (Vumma et al. 2008).

Clozapine’s therapeutic dose in clinical practice is generally assumed to lie between 0.1-1.7 μmol/L (de Leon et al. 2020; Northwood et al. 2023). Previous studies investigating clozapine’s effects on membrane transport of amino acids in human fibroblasts have shown decreased transport of aspartate when fibroblasts were exposed to a supratherapeutic dose of clozapine in the 50 μmol/L range (Marchesi et al. 2006). In another study, 100 μmol/L clozapine, a dose 50 times higher than its therapeutic dose, significantly decreased tyrosine transport during a 1-min uptake period (Bongiovanni et al. 2013). Lithium inhibited transport of tyrosine across cellular membranes in rat brain tissue (Laakso and Oja 1979), and acute administration of lithium decreased tyrosine levels in the striatum of rat brain (McFarlane et al. 2011). Lithium has also been shown to reduce elevated extra-neuronal dopamine levels seen during bipolar mania in animal models of mania (Gambarana et al. 1999; Ichikawa et al. 2005). In the present study, we investigated the effects of clozapine and lithium, alone or in combination on tyrosine transport in skin derived human fibroblasts cultured from healthy controls and bipolar patients. We hypothesized that tyrosine transport in patients with bipolar disorder would be reduced compared to healthy controls, and that either clozapine or lithium or both would restore normal function. We hypothesized also that clozapine and lithium may have different mechanisms of action in this and may have additive or synergistic effects when added together.

## Materials and methods

### Participants

Ten fibroblast cell lines were used in this study. The cell lines originated from forearm skin biopsies from five patients with bipolar disorder type-I (BP-I) and five healthy controls (HC). The cells were stored in a Biobank at the Neuropsychiatric Research Laboratory, School of Medical Sciences, Örebro University. Ethical approval was obtained from the Ethics Committee at the Karolinska Institutet, Stockholm, Sweden (Rnr: 01-205) and Swedish Ethical Review Authority (EPM, 2022-04933-02).

### Cell culture

Fibroblast cell lines from HC (*n* = 5) and BP-I (*n* = 5) between 7 and 16 passages were used in the present study (Table 1). Cells were cultured to confluence at 37 °C and 5% CO_2_ in T75 flasks (Corning Life Sciences, Europe, Netherlands) containing minimum essential medium (MEM) supplemented with 10% FBS, 2 mM L-glutamine, Amino-Max and 100 mg/mL penicillin, and streptomycin (Fisher Scientific GTF AB, Sweden). Radiolabeled [14C] L-tyrosine with the specific activity 482.0 mCi/mmol (Perkin Elmer, USA) and 51.8 mCi/mmol (Larodan Fine Chemicals AB, Sweden) was used to quantify tyrosine uptake in cells seeded in 24 multi-well plates (Corning Life Sciences, USA). All solutions were made with phosphate buffered saline (PBS) (National Veterinary Institute of Sweden, Uppsala (SVA) with pH held at 7.35–7.40.

**Table 1:** Age, gender, and passage number of fibroblast cell lines.

Incubation time	5 min			6 h		
Subjects	HC (*n* = 5)	BP (*n* = 5)	*p*	HC (*n* = 5)	BP ( *n* = 5)	*p*
Age	29.0 ± 7.6	40.0 ± 7.4	0.12	26.0 ± 4.0	43.0 ± 8.0	0.027
Range	(19–41)	(29–50)		(19–31)	(29–50)	
Gender						
Male Female	1 (20%) 4 (80%)	3 (60%) 2 (40%)	0.72	2 (40 %) 3 (60%)	3 (60%) 2 (40%)	0.26
Passage	12 ± 2	11 ± 3	0.87	14 ± 1	11 ± 2	0.08

*HC* healthy controls, *BP* patients with bipolar disorder, *n* number of subjects, *SD* standard deviation. Data was analyzed with independent sample t-test or Chi-square test as found appropriate. HC in the 6-h drug incubation time were younger compared to BP. No other significant difference was noted

### Experimental procedure

Unlike lithium, which has a narrow therapeutic dose interval, the therapeutic window for clozapine varies across a wider range (0.1–1.7 μmol/L) (de Leon et al. 2020; Northwood et al. 2023). To determine optimal clozapine dose within the therapeutic window, a single cell line from a HC was studied at drug incubation times of 5, 30, and 60 min in the absence (baseline) and presence of 0.5, 1.0, and 1.5 μmol/L clozapine. To determine the effects of clozapine and lithium on tyrosine transport, cells were incubated in the absence (baseline) and presence of clozapine, lithium, or combination of both. For each cell line, experiments were carried out twice in triplicates at drug incubation times of 5 min and 6 h under the following conditions: (i) in the absence of psychoactive drugs and in the presence of (ii) 1.0 μmol/L clozapine, (iii) 1 mmol/L lithium, or (iv) 1.0 μmol/L clozapine together with 1 mmol/L lithium.

### Determination of tyrosine uptake

**Fig. 1:**
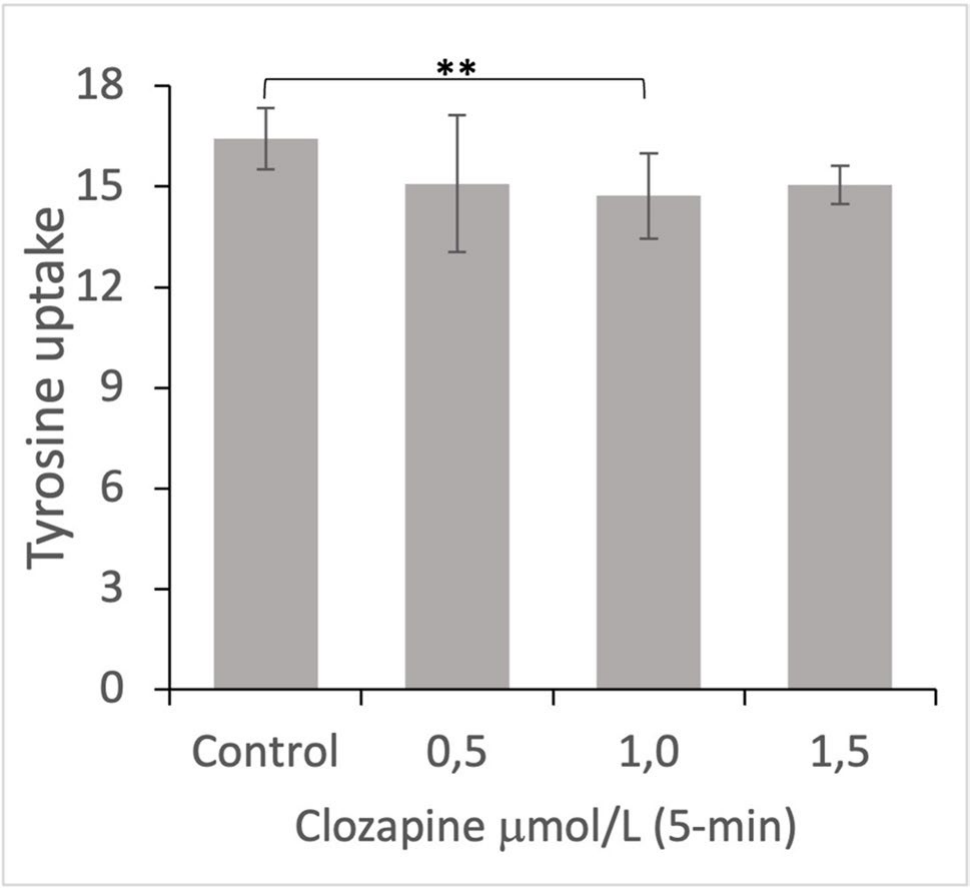
Repeated measures ANOVA, *p*-values Bonferroni adjusted for multiple comparison. Clozapine significantly reduced tyrosine uptake (nmol/5 min × mg protein) in healthy controls at 1.0 μmol/L (*p* = 0.008), but not at 0.5 μ mol/L (p = 0.98). At 1.5 μmol/L, clozapine initially significantly reduced tyrosine uptake by roughly 10%, but this did not survive Bonferroni correction for multiple comparison (*p* = 0.16). ⁎⁎ = *p* ≤ 0.01

Cluster tray method was used to determine tyrosine uptake in fibroblast cell lines (Gazzola et al. 1981) that have been incubated for 5 min or 6 h with saline, clozapine, lithium, or combination of clozapine and lithium. To deplete endogenous amino acid pools, cells were incubated in PBS containing 1% D-glucose solution (Ambresco AB, Ohio, USA) at 37 °C for 1 h. The solution was then removed, and the cells were incubated for 5 min at 37 °C with 200 μl uptake solution always containing 100 μl unlabeled tyrosine (Sigma Aldrich, Germany) and 10 μl [14C] L-tyrosine (Perkin Elmer, USA) and 90 μl PBS that contained (i) no psychoactive drug (baseline), (ii) lithium with a final concentration of 1 mmol/L, (iii) clozapine with a final concentration of 1 μmol/L or (iv) lithium and clozapine at the above final concentrations. Tyrosine uptake was measured at a final tyrosine concentration of 0.1mmol/L. There was thus an additional 5-min incubation time with psychoactive drugs for the 6-h experiments. Cells were then washed twice with ice cold PBS to terminate tyrosine transport into cells, followed by incubation with 0.5 M sodium hydroxide (NaOH) (Fisher Scientific GTF AB Sweden) for 30 min to dissolve the cells. From each well 150 μl of the resolution was removed and mixed with 10 ml scintillation cocktail (Optiphase Hisafe 3, Perkin Elmer, USA), prepared for Liquid scintillation counter (Winspectral 1414, Perkin Elmer, USA) for determination of radioactivity in each sample. The Bradford method was then used to quantify the total amount of protein in each well (Bradford 1976). 20 μl of the solution from each well was transferred to the 96-well plate (Starstedt AG and CO, Germany) followed by bovine serum albumin (Fisher Scientific GTF AB Sweden) used as standard. Multiscan MS (Labsystem Helsinski Finland) was used for determination of protein concentrations (see Fig. 1A, B, Supplementary Information).

### Preparation of clozapine and lithium solutions

Clozapine (Sigma Aldrich, Israel) was dissolved in 1.0 ml dimethyl sulfoxide (DMSO, Sigma Aldrich, Schnelldorf, Germany) to a stock solution of 50 mmol/L and stepwise diluted to final concentrations of 0.5–1.5 μmol/L in phosphate buffer (PBS); final concentration of DMSO was under 0.01%. Lithium sulfate (BioPhausia AB, Stockholm, Sweden) was diluted to a final concentration of 1.0 mmol/L in PBS.

### Statistical analysis

Tyrosine uptake was compared between HC and BP in the absence and presence of the psychoactive drugs clozapine and lithium. Both within-group (lithium/clozapine vs. control conditions in the absence of psychoactive drugs) and between-group (baseline, lithium, or clozapine in HC vs. baseline, lithium, or clozapine in BP) pairwise comparisons were conducted. Kolmogorov-Smirnov test showed data was not always normally distributed, thus parametric statistics with independent sample *t*-test and one-way ANOVA or nonparametric test with independent samples Mann-Whitney *U* or independent samples Kruskal-Wallis test were used as found appropriate. Where significant interaction between group and medication was found, post-hoc test was performed to determine differences. SPSS (IBM v28) was used for all statistical analysis and *p*-value ≤ 0.05 after Bonferroni correction for multiple comparison was considered significant. All significant values shown are for two-sided tests.

## Results

### Characteristics of fibroblast cell lines

Ten fibroblast cell lines were randomly selected from a biobank, 5 healthy controls (HC), and 5 patients with bipolar disorder (BP). Mean age, gender distribution, and number of passages for both HC and BP is shown in Table 1. HC were significantly younger than BP, but no other difference was found significant.

### Determination of optimal clozapine dose

Lithium which has a narrow therapeutic window (0.5–1.2 mmol/L) was used at a concentration of 1 mmol/L, which is at the upper end of its therapeutic dose interval. To determine optimal dose for clozapine within its wider therapeutic range, preliminary experiments were conducted in a single cell line from a healthy donor incubated with saline or 0.5, 1.0, and 1.5 μmol/L clozapine for 5, 30, or 60 min (*n*=6 at each incubation time). Repeated measures ANOVA showed significant main effect for condition for the 5-min (*p* = 0.016, ηp^2^ 0.70), but not for the 30-min (*p* = 0.39) or 60-min (*p* = 0.16) incubation times. Bonferroni corrected pairwise comparison for the 5-min experiments showed significant clozapine effect at 1.0 μmol/L, which decreased tyrosine uptake by roughly 10% (*p* = 0.008, Fig. 1). There was also a roughly 10% decrease in tyrosine uptake at both 0.5 and 1.5 μmol/L that did not however remain significant after Bonferroni correction (*p* = 0.98 and 0.14, respectively). Although there was no main effect for condition for the 60-min incubation time, post-hoc pairwise comparison showed significant effect of clozapine at 1.0 μmol/L (−14.3%; *p* = 0.023) and 1.5 μmol/L (−10.2%; *p* = 0.05), which again did not survive Bonferroni correction for multiple comparison.

### Baseline tyrosine uptake in the absence of psychoactive drugs

**Fig. 2:**
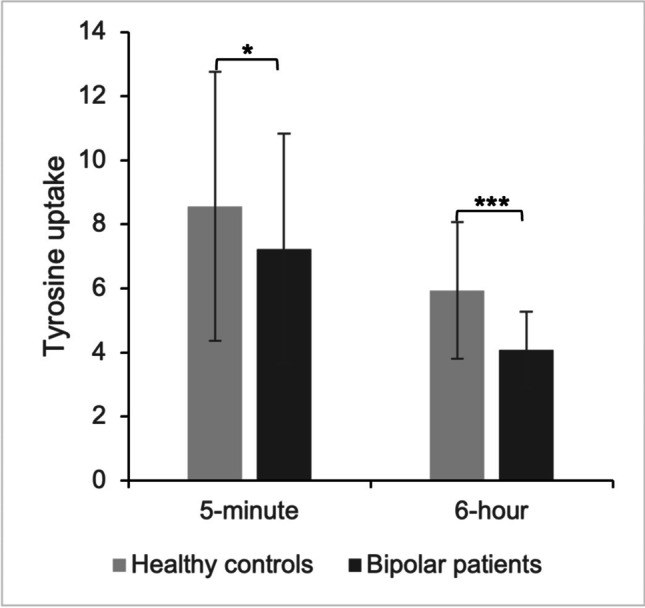
Tyrosine uptake (nmol/5 min x mg protein) in the absence of psychoactive drugs at 5-min and 6-h drug incubation times. Mann-Whitney *U* test showed significant deficit in bipolar patients compared to healthy controls for both the 5-min (−15.5%; *p* = 0.044) and 6-h (−31.2%; *p* < 0.001) incubation times. Shown is median ± IQR. The 6-h but not the 5-min drug incubation time survived Bonferroni correction for multiple comparison

To compare tyrosine uptake between HC and BP in the absence of psychoactive drugs, fibroblast cell lines were incubated in MEM diluted with 1% FBS for 5 min (*n* = 24 for HC and *n* = 20 for BP) or 6 h (*n* = 28 for both HC and BP). At 5-min incubation time, BP had 15.5% lower tyrosine uptake compared to HC (Md 8.56, IQR 4.19 and Md 7.23, IQR 3.59 nmol/5 min × mg protein, respectively; Mann-Whitney *U* test, *p* = 0.022). The difference in tyrosine uptake between HC and BP grew with increasing incubation time and there was roughly 31.2% lower tyrosine uptake in BP compared to HC at 6-h incubation time (Md 5.93, IQR 2.13 and Md 4.08, IQR 1.19 nmol/5 min × mg protein, respectively; Mann-Whitney *U* test, *p* < 0.001). The difference at 6-h, but not that at 5-min incubation time, survived Bonferroni correction (Fig. 2).

### The effect of clozapine and lithium on tyrosine uptake

**Fig. 3:**
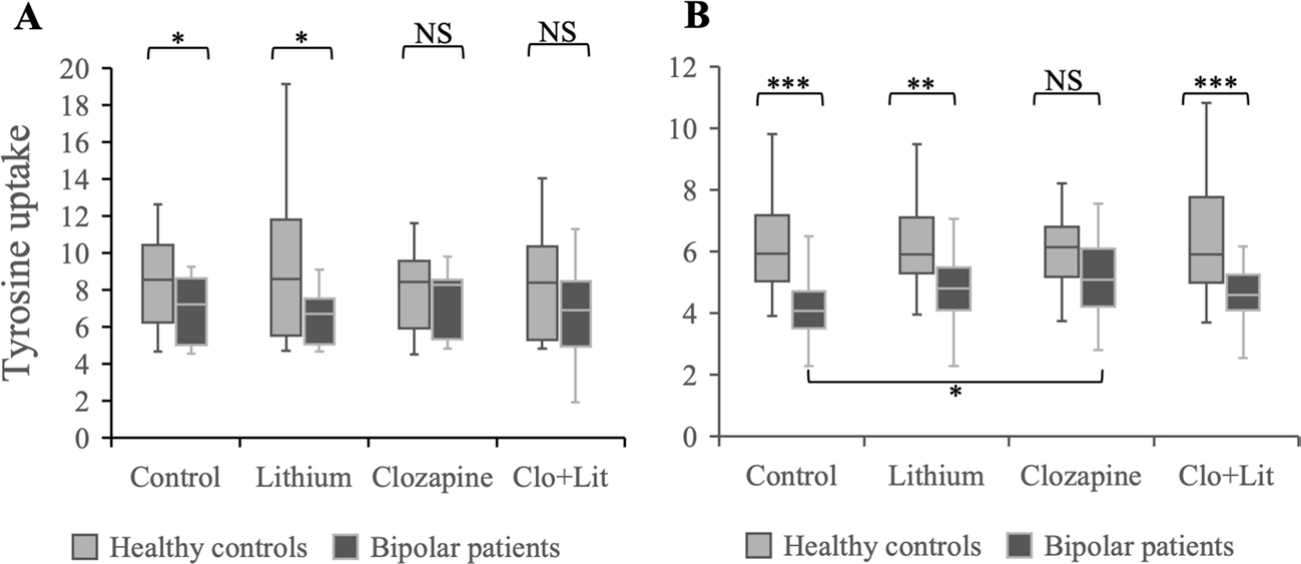
Tyrosine uptake (nmol/5 min × mg protein) in healthy controls and bipolar patients under different conditions as indicated during 5-min (**A**) and 6-h (**B**) drug incubation times. Shown is median ± IQR. Kruskal-Wallis test with *p*-values adjusted for multiple comparison with Bonferroni correction: NS = not significant; * = *p* < 0.05; ** = *p* < 0.01; *** = *p* < 0.001. Bipolar patients had significantly lower tyrosine uptake than healthy controls except in the presence of clozapine. The initial between-group differences seen at 5-min incubation time (**A**) did not survive Bonferroni correction for multiple comparison. Clo+Lit = Clozapine+Lithium

To study immediate and sustained effects of psychoactive drugs on tyrosine uptake in the two groups (HC vs. BP) under four different conditions (baseline, clozapine, lithium, and clozapine + lithium), therapeutic doses of 1.0 μmol/L clozapine, 1.0 mmol/L lithium, or a combination of both at the above concentrations was added to the incubation medium for 5 min or 6 h. Independent samples Kruskal Wallis test showed significant main effect for group (HC vs. BP) in both the 5-min (H = 17.2(7, *N*=117), *p* = 0.016) and 6-h (H = 60.5(7, N=228), *p* < 0.001) incubation times (Table 2), and a main effect for condition (baseline × clozapine × lithium × clozapine + lithium) for the 6-h incubation time in BP (H = 8.26(3, *N*=117), *p* = 0.035) but not HC (H = 0.34(3, *N*=108), *p* = 0.95). Bonferroni adjusted post-hoc within-group analysis in BP showed significant effect for clozapine compared to baseline (*p* = 0.026), but not for lithium (*p* = 0.24) or combination of clozapine and lithium (*p* = 0.18) (Table 3, Fig. 3B). In the 5-min incubation time, there was no within-group main effect for condition, neither in HC (H = 0.88(3, *N*=92), *p* = 0.83) nor in BP (H = 3.1(3, *N*=81), *p* = 0.39), although there was a similar trend with clozapine enhancing tyrosine uptake in BP and reversing the difference between HC and BP seen under baseline conditions.

**Table 2:** The effect of clozapine, lithium and clozapine + lithium (Clo+Lit) on tyrosine uptake in healthy controls and patients with bipolar disorder during the 6-h drug incubation time. There was no main effect of medication in healthy controls, thus no post-hoc analysis was conducted

Kruskal-Wallis and within-group post-hoc analysis (compared to baseline in each group)										
Healthy controls No main effect for medication						Bipolar patients Kruskal-Wallis H(117,3) 8.62, *p* = 0.035				
	*n*	Md	IQR	*p*	*p*-corr	*n*	Md	IQR	*p*	*p*-corr
Baseline	28	5.93	2.13	–	–	28	4.08	1.19	–	–
Clozapine	26	6.14	1.81	NA	NA	29	5.06	1.43	0.004	0.025
Lithium	27	5.91	1.63	NA	NA	30	4.81	1.88	0.041	0.247
Clo + Lit	27	5.92	2.78	NA	NA	30	4.58	1.05	0.122	0.731

*n* number of experiments, *Md* median, *IQR* interquartile range, *NA* not analyzed; *p*-*corr p*-values corrected for multiple comparison with Bonferroni

**Table 3:** Comparison of tyrosine uptake between healthy controls (HC) and patients with bipolar disorder (BP) at baseline (bl) and in the presence of clozapine (clo), lithium (lit), and clozapine + lithium (clo+lit)

Kruskal-Wallis and between-group post-hoc analysis (compared at each condition)								
	5-min drug incubation time Kruskal-Wallis H(173,7) 17.1, *p* = 0.016				6-h drug incubation time Kruskal-Wallis H(225,7) 60.5, *p* <0.001			
	*n*	*Z*	*p*	*p*-corr	*n*	*Z*	*p*	*p*-corr
HC-bl vs. BP-bl	24, 20	2.30	0.021	0.126	28, 28	4.76	<0.001	0.0001
HC-clo vs. BP-clo	22, 19	0.99	0.321	0.99	27, 27	2.45	0.014	0.084
HC-lit vs. BP-lit	24, 21	2.59	0.01	0.06	26, 26	3.57	<0.001	0.002
HC-clo+lit vs. BP-clo+lit	22, 21	1.75	0.08	0.48	27, 27	3.96	<0.001	0.0004

*Md* median, *IQR* interquartile range; *p-corr p*-values corrected for multiple comparison with Bonferroni

Between-group pairwise post-hoc analysis of HC and BP under the different experimental conditions is shown in Table 3 and illustrated in Fig. 3 A and B. The reuslts showed that the group differences seen at baseline between HC and BP both during the 5-min and 6-h incubation time (*p* = 0.016 and *p* < 0.001, respectively) remained significant for lithium (*p* = 0.002) and combination of clozapine and lithium (*p* = 0.00046), but not for clozapine (*p* = 0.084) at 6-h incubation (Fig. 3A, B; Table 3). In other words, the differences between HC and BP seen under baseline conditions were abolished by clozapine but not by lithium. The between-group differences at 5-min incubation time did not, however, survive Bonferroni correction for multiple analysis.

## Discussion

Clozapine and lithium have unique place in the treatment of psychotic and affective disorders. Both drugs are first-line options for treatment-resistant schizophrenia and bipolar disorder, respectively. Clozapine is also known to have mood-stabilizing effects (Kapczinski et al. 2021; Okhuijsen-Pfeifer et al. 2018) and lithium is used as adjunctive treatment in schizoaffective disorder, which has both psychotic and affective components. Within their respective therapy domains, both drugs are also first-line treatment options in patients with high risk for suicide (Saunders and Hawton 2009) and chronic aggressive behavior (Delgado et al. 2020; Foster 2013; Frogley et al. 2012; Kelly et al. 2006). Currently, it is not known whether clozapine and lithium reduce suicidal behavior only within their therapeutic domains or have wider transdiagnostic effects irrespective of the underlying disorder (Al Jurdi et al. 2015). It is not known either whether they have additive or synergistic effects in reducing suicidal and aggressive behavior. Based on clinical experience, combination treatment with clozapine and lithium is increasingly being used in managing patients with severe self-harm and suicidal behavior (Delgado et al. 2020; Fornaro et al. 2020; Frogley et al. 2012; Kelly et al. 2006; Li et al. 2015), under the tacit assumption that their anti-suicide effects are additive or at the very least not antagonistic. Another area where clozapine and lithium are used in combination is when clinicians try to mitigate the known adverse effect of clozapine, agranulocytosis, by taking advantage of lithium’s diametrically opposite effect in causing leukocytosis (Boazak et al. 2018). Identifying cellular processes modified by clozapine and lithium in an additive or synergistic manner would therefore aide in zeroing in on their mechanism of action in reducing suicidal behavior.

Tyrosine is a precursor of dopamine and noradrenaline, whose conversion to L-DOPA by tyrosine hydroxylase is the rate-limiting step in the synthesis of the two signal substances. Dopamine and noradrenaline are known to have widespread cognitive and affective functions (Berridge and Arnsten 2013) and are implicated in the pathogenesis of various psychiatric ailments, including ADHD, psychosis, affective disorders, and substance abuse syndrome (Arnsten 2007; Grinchii and Dremencov 2020; Xing et al. 2016). Dopamine and noradrenaline are also in one way or the other implicated in the pharmacological treatment of most psychiatric disorders, as numerous psychoactive drugs including clozapine and lithium act by modifying the function of these two signal substances. In the present paper, we studied the effects of therapeutic doses of clozapine and lithium on tyrosine transport across fibroblast cell membrane with the following aims: (i) to replicate previous findings (Persson et al. 2009) reporting significant deficit in tyrosine uptake in patients with bipolar disorder compared to healthy controls in the absence of psychoactive agents, (ii) to see if clozapine or lithium or both can reverse this deficit and normalize tyrosine transport, and (iii) provided that one or both have effect, to see whether this is additive, synergistic or antagonistic when both are added together.

Fibroblasts cultured from patients with different diagnosis have previously been used to study pathological mechanisms of psychiatric disorders (Batalla et al. 2015; Flyckt et al. 2001; Garbett et al. 2015; Kálmán et al. 2016; Persson et al. 2009), and also to study cellular mechanisms of psychoactive agents (Seshadri et al. 2017; Viswanath et al. 2015). In the absence of psychoactive drugs, tyrosine uptake in fibroblast from patients with bipolar disorder was previously shown to be reduced compared to healthy controls (Persson et al. 2009). Persson et al. (2009) studied the kinetics of tyrosine uptake at different concentrations using a 1-min tyrosine incubation and found significantly reduced uptake capacity in patients with bipolar disorder compared to healthy controls. In the present study, when we studied tyrosine uptake at a fixed concentration (0.1 mmol/L) and 5-min tyrosine incubation, we found a 30% reduction in tyrosine uptake, and that clozapine but not lithium reversed this deficit. Thus, while the methodologies we used in the present study were somewhat different from those of Persson et al., (2009) our results were internally consistent. Clozapine appeared to have a diagnosis specific effect on tyrosine transport, with a trend towards double dissociation at 5-min incubation time — with small but significant enhancement of tyrosine uptake in bipolar patients (Fig. 3A, B) and an equally small but significant reduction in healthy controls (Fig. 1). Both of this effects did not, however, survive Bonferroni correction for multiple comparisons. Clozapine had also diagnosis specific effect on tyrosine transport at 6-h incubation time, i.e., selectively and significantly enhancing tyrosine uptake in bipolar patients but not in healthy controls. This later effect was robust and readily survived Bonferroni correction for multiple comparison. These findings strongly suggest that clozapine has little or no effect on tyrosine transport under physiological conditions, but robustly enhances it under pathophysiological conditions.

### Combination treatment with clozapine and lithium

Contrary to our expectation, we saw no additive or synergistic effects of clozapine and lithium on tyrosine uptake when the two drugs were added together. To the contrary, clozapine was less effective in modifying tyrosine transport when added together with lithium than when added alone. One possible explanation for this could be that the two drugs competed for binding at some putative common site of action, and clozapine in the presence of lithium could not bind well enough to reach the required threshold for action. Irrespective of possible mechanisms behind this, we believe this finding may have important implications in the clinical setting where polypharmacy is routinely utilized. Combination treatments might under certain circumstances lead to less optimal outcome, not just by increasing risk for adverse events or through pharmacokinetic interactions, but also through pharmacodynamic interactions even when the medications do not have any known common mechanism of action.

### Potential clinical implications

Tyrosine transport across the blood brain barrier occurs through the LAT1 and LAT2 transporters, both of which are expressed in fibroblasts. Thus, fibroblast cell lines collected from healthy donors and patients with bipolar disorder can be used as surrogate models for the study of tyrosine uptake in the CNS. Tyrosine supplementation has also been shown to have cognition enhancing effects under stressful and demanding conditions when dopaminergic neurons are actively releasing transmitter substance (Jongkees et al. 2015). A correlation between baseline cognitive capacity and dopamine synthesis, and by extension tyrosine availability, has also been demonstrated (Cools et al. 2008). Studies on individuals at high-risk for developing psychosis have shown important role for dopamine synthesis (and by extension tyrosine availability), such that those high-risk individuals who transitioned to psychosis had significantly higher dopamine synthesis capacity compared to healthy controls and to high-risk individuals who did not develop psychosis (Howes et al. 2011). In a similar study that further extended the findings of Howes et al. (2011), Avram et al. (2019) found that schizophrenia patients who had remitted from psychotic symptoms had reduced dopamine synthesis capacity compared to healthy controls, and that this difference was correlated to cognitive impairment (Avram et al. 2019). Dopamine synthesis being dependent on tyrosine availability, the tyrosine uptake deficit in bipolar patients we demonstrate in the present study at baseline, which is normalized by clozapine but not lithium can thus be expected to have real-world clinical implications in patient populations.

The mechanism of action of clozapine as mood stabilizer is not known, while its antipsychotic effects at least in part is assumed to be related to dopamine D2 blockade (Lieberman et al. 2008). The findings of the present study indicate that clozapine may increase dopamine synthesis by normalizing tyrosine transport deficit seen in bipolar patients in the present study and in Persson et al. (2009), as well as that previously described in patients with schizophrenia (Flyckt et al. 2001; Hagenfeldt et al. 1987; Ramchand et al. 1996). Studies in animal models have also previously shown that clozapine increases DA outflow in the cortex (Invernizzi et al. 1990; Moghaddam and Bunney 1990). In discussing potential clinical relevance of the present findings, focus will be put on clozapine’s anti-psychotic effects, which is just as important in schizophrenia as it is in bipolar disorder in the presence of affective psychosis. There are different neurochemical models for psychosis, including dopaminergic, glutamatergic, and serotonergic models (Yang and Tsai 2017). The dopamine hypothesis posits that positive symptoms (delusions and hallucinations) are caused by D2-receptor hyperactivity in the mesolimbic dopamine pathway and negative symptoms and cognitive impairment due to D1-receptor hypoactivity in the mesocorticolimbic pathway (Abi-Dargham et al. 2002). Currently available antipsychotic medications are generally effective in reducing positive symptoms, but largely ineffective in modifying negative symptoms and cognitive impairment. Clozapine is one of the few antipsychotic agents believed to have efficacy on both positive and negative schizophrenia symptoms. The effect on positive symptoms is generally believed to be mediated through D2-receptor blockade, but there is less clarity on how clozapine could improve negative symptoms. Possible explanation often given is that second generation antipsychotics (SGA), including clozapine, do not really improve primary negative symptoms, but appear to do so because SGA unlike first generation antipsychotics (FGA) do not cause secondary negative symptoms. One implication of our finding is that clozapine may differentially address D1-receptors (to improve negative symptoms), and D2-receptors (to improve positive symptoms). If the enhanced tyrosine uptake caused by clozapine translates into increased dopamine synthesis, there will be more dopamine available for release that will lead to increased dopamine levels around D1- and D2-receptors. Because 40–50% of D2 receptors will be blocked by clozapine (Wiesel et al. 1990), the increased dopamine release will not lead to exacerbation of psychosis, while D1-receptors will be exposed to higher dopamine levels and clozapine may thus simultaneously address both positive and negative symptoms. Chronic lithium administration has been reported to increase tyrosine levels in rat brain tissue (Jope et al. 1989). In our experiments, there was a weak initial positive effect of lithium on tyrosine uptake in BP, which however did not survive Bonferroni correction for multiple comparison, which leads us to conclude that under our experimental conditions, lithium had no significant effects on tyrosine uptake, but antagonized clozapine effects when both were added together.

### Limitations

Although clozapine is known to have mood stabilizing effects, its main area of use is in schizophrenia and related psychotic disorders. Before we can draw any conclusion pertaining to clozapine’s antipsychotic mechanism, these experiments need to be replicated in fibroblasts from patients with schizophrenia. Furthermore, although fibroblasts express similar amino acid transporter proteins as those found in the blood-brain barrier and neuronal membranes, these may differ in function so the findings here cannot simply be extrapolated to also apply to neuronal tissue. Another limitation that needs to be mentioned here is that there was significant age difference between HC and BP in our study, which may have contributed to the differences observed in tyrosine uptake between HC and BP.

## Conclusions

Tyrosine uptake was reduced in BP compared to HC under baseline conditions, a deficit that was normalized by clozapine but not lithium. Clozapine selectively enhanced tyrosine uptake in BP but slightly reduced it in HC, indicating that it had little effect on tyrosine transport under physiological conditions, but restored function under pathological conditions. Not only was there no additive or synergistic effect in this, but clozapine was no longer as effective when added together with lithium, indicating that medications that may initially have been effective when used alone may no longer be effective when used together with other medications, which we believe may have implications for polypharmacy in clinical settings. Here, it should be remembered, however, that in clinical practice clozapine and lithium are being increasingly used together, in some cases to mitigate the known side effect of clozapine in causing neutropenia, and in other cases to optimize treatment of patients with severe self-harm, and it is not generally believed that lithium and clozapine are clinically more effective when used alone than when combined together.

## Supplementary information

ESM 1
