# Supplementary material for: Clozapine but not lithium reverses aberrant tyrosine uptake in patients with bipolar disorder
Source: Psychopharmacology (Berl). 2023 Jun 15;240(8):1667–76. doi: 10.1007/s00213-023-06397-5 (PMC10349740; doi:10.1007/s00213-023-06397-5)
Supplement: Supplementary file 1 — ESM 1 [file 213_2023_6397_MOESM1_ESM.docx]

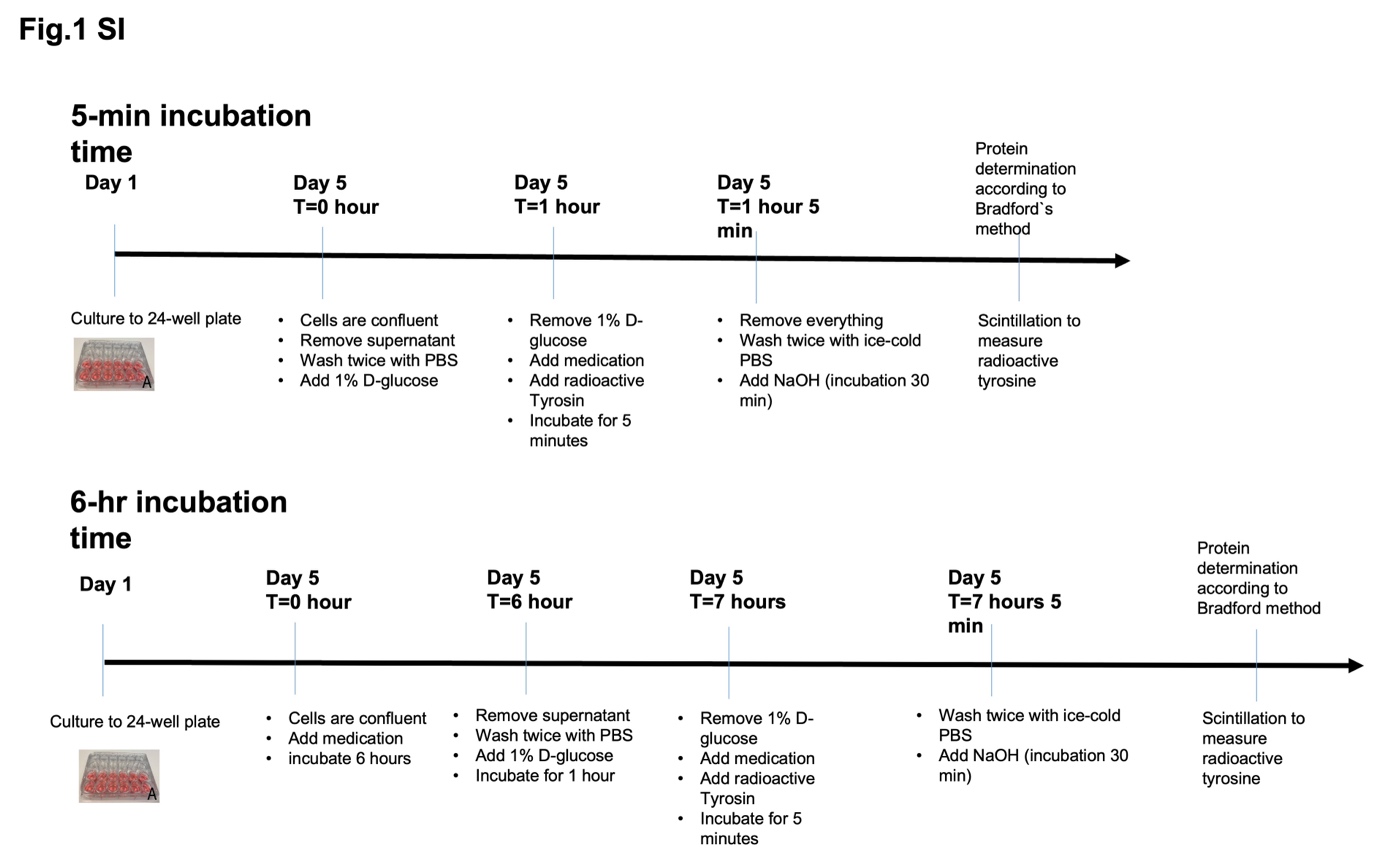


**Fig.1_SI** Timeline for the 5-minute and 6-hour experiments as indicated

**2A 2B**


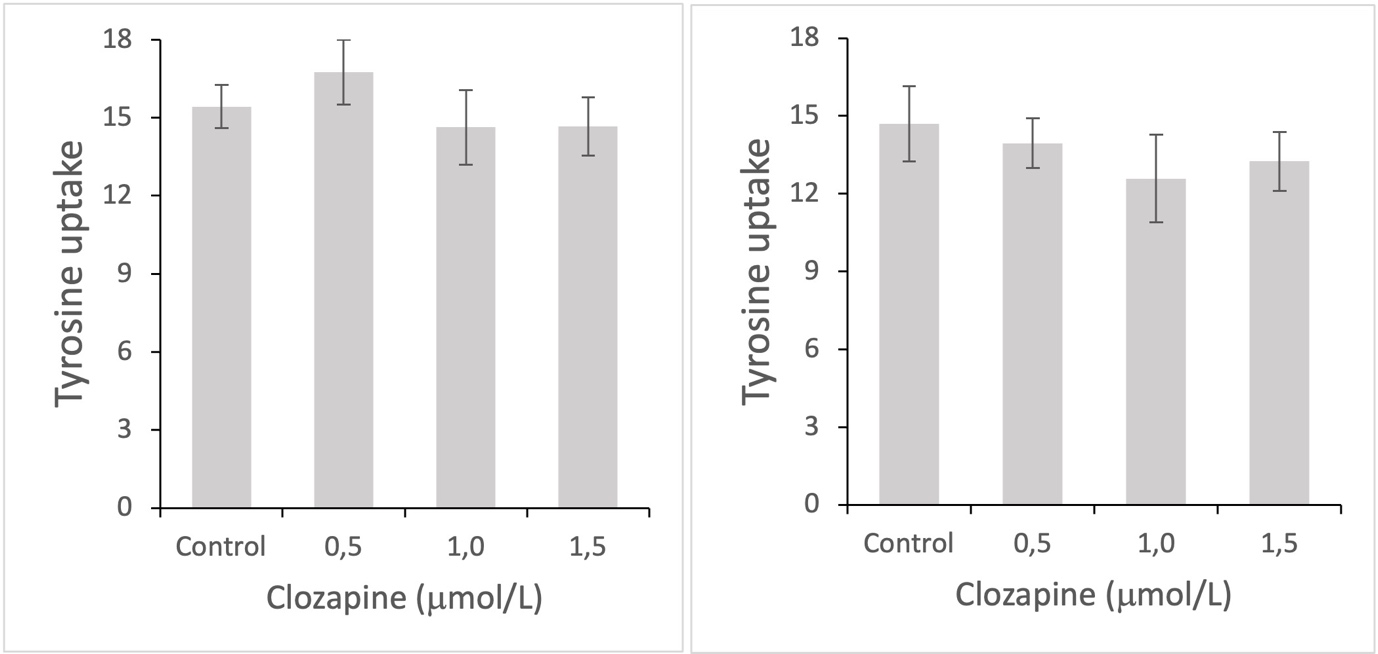


**Fig. 2A-B SI** Tyrosine uptake (nmol/5 min x mg protein) in healthy controls at 0.5, 1.0 and 1.5 μmol/L during incubation time of 30 minutes (2A) and 60 minutes (2B) at different concentrations of clozapine as indicated. Although there was no main effect for condition for both the 30 and 60-min incubation times, post-hoc pairwise comparison during the 60-min incubation time showed significant effect of clozapine at 1.0 μmol/L (-14.3%; p = 0.023) and 1.5 μmol/L (-10.2%; p = 0.05), which did not however survive Bonferroni correction for multiple comparison.

| Kruskal-Wallis and within-group post-hoc analysis (compared to baseline in each group)  at 5-min incubation time | | | | | | | | | | |
| --- | --- | --- | --- | --- | --- | --- | --- | --- | --- | --- |
| Healthy controls  No main effect for medication | | | | | | Bipolar patients  No main effect for medication | | | | |
|  | n | Md | IQR | *p* | *p*-corr | n | Md | IQR | *p* | *p*-corr |
| Baseline | 24 | 8.56 | 4.19 | NA | NA | 20 | 7.23 | 3.59 | NA | NA |
| Clozapine | 22 | 8.44 | 3.66 | NA | NA | 19 | 8.3 | 3.2 | NA | NA |
| Lithium | 24 | 8.58 | 6.31 | NA | NA | 21 | 6.71 | 2.48 | NA | NA |
| Clo + Lit | 22 | 8.41 | 5.05 | NA | NA | 21 | 6.93 | 3.52 | NA | NA |

**Table 1 SI** Effect of clozapine, lithium and clozapine + lithium on tyrosine uptake in healthy controls and patients with bipolar disorder during 5-min incubation time. There was no main effect of medication in healthy controls or bipolar patients, thus no post-hoc analysis was conducted. Md=median; IQR = interquartile range; NA = not analyzed; *p*-corr = p-values corrected for multiple comparison with Bonferroni.
